# Amnion-Derived Mesenchymal Stromal/Stem Cell Paracrine Signals Potentiate Human Liver Organoid Differentiation: Translational Implications for Liver Regeneration

**DOI:** 10.3389/fmed.2021.746298

**Published:** 2021-09-23

**Authors:** Antonio Lo Nigro, Alessia Gallo, Matteo Bulati, Giampiero Vitale, Diego Sebastian Paini, Mariangela Pampalone, Daniele Galvagno, Pier Giulio Conaldi, Vitale Miceli

**Affiliations:** ^1^Ri.MED Foundation, Palermo, Italy; ^2^Research Department, Mediterranean Institute for Transplantation and Advanced Specialized Therapies (IRCCS ISMETT), Palermo, Italy

**Keywords:** 3D liver organoid culture, hepatocyte culture, human amnion-derived mesenchymal stem cells, liver regeneration, hepatic progenitor cell differentiation

## Abstract

The prevalence of end-stage liver diseases has reached very high levels globally. The election treatment for affected patients is orthotopic liver transplantation, which is a very complex procedure, and due to the limited number of suitable organ donors, considerable research is being done on alternative therapeutic options. For instance, the use of cell therapy, such as the transplantation of hepatocytes to promote liver repair/regeneration, has been explored, but standardized protocols to produce suitable human hepatocytes are still limited. On the other hand, liver progenitor and multipotent stem cells offer potential cell sources that could be used clinically. Different studies have reported regarding the therapeutic effects of transplanted mesenchymal stromal/stem cells (MSCs) on end-stage liver diseases. Moreover, it has been shown that delivery of MSC-derived conditioned medium (MSC-CM) can reduce cell death and enhance liver proliferation in fulminant hepatic failure. Therefore, it is believed that MSC-CM contains many factors that probably support liver regeneration. In our work, we used an *in vitro* model of human liver organoids to study if the paracrine components secreted by human amnion-derived MSCs (hAMSCs) affected liver stem/progenitor cell differentiation. In particular, we differentiated liver organoids derived from bipotent EpCAM^+^ human liver cells and tested the effects of hAMSC secretome, derived from both two-dimensional (2D) and three-dimensional (3D) hAMSC cultures, on that model. Our analysis showed that conditioned medium (CM) produced by 3D hAMSCs was able to induce an over-expression of mature hepatocyte markers, such as ALB, NTCP, and CYP3A4, compared with both 2D hAMSC cultures and the conventional differentiation medium (DM). These data were confirmed by the over-production of ALB protein and over-activity of CYP3A4 observed in organoids grown in 3D hAMSC-CM. Liver repair dysfunction plays a role in the development of liver diseases, and effective repair likely requires the normal functioning of liver stem/progenitor cells. Herein, we showed that hAMSC-CM produced mainly by 3D cultures had the potential to increase hepatic stem/progenitor cell differentiation, demonstrating that soluble factors secreted by those cells are potentially responsible for the reaction. This work shows a potential approach to improve liver repair/regeneration also in a transplantation setting.

## Introduction

Acute and chronic liver diseases are important illnesses affecting morbidity and mortality globally, and the underlying causes vary across different countries and ethnic groups (1). The management of these patients is difficult and the gold standard for the treatment is orthotopic liver transplantation (OLT) in the case of organ failure. However, the shortage of suitable donor organs defines an imbalance between the number of patients on the waiting list and available organs (2), and that has led to an increase in the mortality of patients on the waiting list for OLT (3). In this scenario, some strategies are emerging in order to expand the donor pool, which involves the use of both living donors (4) and marginal grafts (5). Moreover, cell-based therapies are also emerging to treat liver diseases (6). Indeed, various groups have used hepatocyte transplants to provide organ support/regeneration and promote liver function in patients with liver failure (7–9). However, the use of fresh human hepatocytes has constraints such as organ availability, limited cell proliferation and function, and the risk of immune rejection (10). In addition, the use of cryopreserved human hepatocytes was restricted by the instability of hepatocyte cultures and the lack of standardized protocol for hepatocyte expansion, which quickly de-differentiates and therefore limits their application. On the other hand, liver progenitor and multipotent stem cells offer potential cell sources that could be used clinically for the treatment of liver diseases (11). Cell-based therapies provide a promising strategy to support liver regeneration (12) and could represent a hopeful approach to improve solid organ transplantation in patients with organ failure. Furthermore, cell therapy could also delay disease progression and facilitate a more aggressive resection of the liver in patients with hepatocellular carcinoma.

In order to make liver cell transplantation a safer and useful procedure to treat end-stage liver diseases, new cognizance is needed on the mechanisms underlying liver regeneration/differentiation, where hepatic progenitor cells play a crucial role. In recent years, three-dimensional (3D) culture systems have been developed to generate tissue-like structures called organoids. Organoids are *in vitro* cellular cultures that allow cells to self-organize, replaying the structure/function of the *in vivo* tissue, and provide a useful system in both clinical and basic research (13). Many kinds of organoid models have been obtained from primary cells (14) including bipotent stem cells (EpCAM^+^ cells) derived from adult human liver (15). Here, the cells allow mimicking *in vitro* what happens during liver embryonic development, where embryonic liver cells (derived from endoderm) change into a non-polarized cellular phenotype called hepatoblasts. These hepatoblasts are bipotent cells expressing fetal liver genes and protein associated with both hepatocyte and cholangiocyte lineages and can differentiate into both cell types (16). Therefore, the ability to culture functional hepatocytes from bipotent liver progenitor cells represents a great opportunity to directly study the mechanisms of human liver disease, perform high-throughput drug screening for new therapies, and facilitate hepatocyte transplantation (17–19).

Matsumoto et al. showed that during embryonic development, surrounding mesenchymal stromal/stem cells (MSCs) have a crucial role in the formation of the liver primordium from the endoderm (20). MSCs could control the liver progenitor cells and regulate their fate by creating an embryonic niche that promotes liver development. Recreating this embryonic niche represents a promising *in vitro* approach to understanding how to improve hepatic differentiation and translate this knowledge into clinical practice. Progenitor liver cells cultured as 3D liver organoids in the presence of MSCs might allow for further hepatic differentiation, but limited information is available about the mechanisms by which MSCs regulate hepatic differentiation. In this study, we hypothesized that MSC-derived soluble factors, not depending on cell–cell contact, regulate hepatic differentiation of liver progenitor cells. To test this hypothesis, we first performed an *in vitro* culture system to differentiate liver progenitor cells into mature hepatocyte-like cells. We then implemented the spheroid culture method for MSCs to increase the production of their paracrine factors, and we used MSC secretome to enhance liver organoid differentiation.

## Materials and Methods

### Cell Cultures

HepG2 and HDFa cells were obtained from the American Type Culture Collection (ATCC, USA). Cells were routinely grown and maintained in Roswell Park Memorial Institute (RPMI) medium (GIBCO, USA) (HepG2) or Dulbecco's modified Eagle's medium (DMEM) (GIBCO, USA) (HDFa), supplemented with 10% fetal bovine serum (FBS) (Thermo Fisher Scientific, USA), 2 mmol/l L-glutamine, 100 U/ml penicillin, and 100 μg/ml streptomycin (GIBCO, USA) at 37°C with 5% CO_2_.

### Human Liver Organoid Expansion

Liver biopsies were obtained during liver transplantation in accordance with the ethical standard of the 1975 Declaration of Helsinki. Informed consent was obtained from each donor. Written informed consent and details of the procedure were approved by the Institutional Research Review Board of the Institute of the Mediterranean for Transplantation and High Specialty Therapies (ISMETT) (project identification code: IRRB/13/17). Liver cell suspension was obtained from biopsies after digestion with collagenase. Briefly, tissue was minced and washed two times with DMEM supplemented with 1% FBS. It was then incubated with the digestion solution (2.5 mg/ml collagenase D, 0.1 mg/ml DNase I) (Roche, Germany) in Earle's balanced salt solution (EBSS) (Thermo Fisher Scientific, USA) for 20–40 min at 37°C. The digestion was stopped by adding cold DMEM (supplemented with 1% FBS) and the suspension was filtered through a 70 μm cell strainer (BD Falcon, USA). Cells were then washed two times with phosphate-buffered saline (PBS) and resuspended in PBS supplemented with 0.01% NaN_3_ (Sigma, USA) and 5% normal mouse serum (Thermo Fisher Scientific, USA) for CD326-APC (EpCAM) labeling and subsequent separation by fluorescence-activated cell sorting (FACS) with FACSAria flow cytometer (BD Biosciences, USA). EpCAM^+^ cells were pelleted by centrifugation at 300 g for 5 min and washed with cold advanced DMEM/F12 (AdDMEM/F12) (GIBCO, USA). The cell pellets were mixed with basement membrane extract, type 2, Pathclear (BME 2, AMSBIO, UK), and 3,000–6,000 cells were seeded per well in a 48-well plate (BD Falcon, USA). After BME 2 had solidified, culture medium for organoid expansion (expansion medium, EM) was added. This medium was made with AdDMEM/F12 supplemented with 1% glutamax (GIBCO, USA), 10 mM 4-(2-hydroxyethyl)-1-piperazineethanesulfonic acid (HEPES) (GIBCO, USA), 1% of both N2 and B27 supplements (without vitamin A) (GIBCO, USA), 1.25 mM N-Acetylcysteine (Sigma, USA), 10 mM Nicotinamide (Sigma, USA), 10 nM gastrin (Sigma, USA), 50 ng/ml epidermal growth factor (EGF) (Peprotech, UK), 100 ng/ml fibroblast growth factor 10 (FGF10) (Peprotech, UK), 25 ng/ml hepatocyte growth factor (HGF) (Peprotech, UK), 25 ng/ml Noggin (Peprotech, UK), 500 ng/ml RSPO1 (MedChemExpress, USA), 5 μM A83-01 (MedChemExpress, USA), 0.5 μM CHIR99021 (MedChemExpress, USA), 10 μM Forskolin (MedChemExpress, USA), and 10 μM Y27632 (MedChemExpress, USA). Organoids were cultured for 10–14 days, mechanically dissociated into small fragments, and splitted (1:4–1:8) in fresh BME 2 every 7–10 days. In addition, the cultures were dissociated by incubation with TrypLE (GIBCO, USA) until single cell suspension and cell numbers were counted by trypan blue exclusion.

### Immunofluorescence of Liver Organoids

Liver cell suspension (EpCAM^+^ cells) was mixed with BME 2 (AMSBIO, UK), and 3,000–4,000 cells were seeded per well in chambered cell-culture slides (8-well, Corning, USA). Following the formation of organoids, cells were fixed in 4% paraformaldehyde for 30 min and then permeabilized with 0.1% Triton PBS for 15 min at room temperature. The slides were incubated with blocking buffer (PBS/BSA 1%, 2.5 mM EDTA, 5% immunopure normal goat serum, Thermo Fisher Scientific, USA) for 1 h at room temperature and then with primary rabbit monoclonal antibodies against epithelial cell adhesion molecule (EpCAM) (1:100 dilution, Abcam, UK) overnight at 4°C. After three washes with PBS, slides were incubated with goat anti-rabbit IgG secondary antibody (Alexa Fluor® 488, Abcam, UK) at 1/1,000 dilution for 2 h at room temperature. After three washes with PBS, coverslips were then mounted on slides using a fluorescent mounting medium with 4′ 6-diamidino-2-phenylindole (DAPI) (Thermo Fisher Scientific, USA), and samples were visualized under a Leica confocal station (Leica SP5 confocal system) mounted on a Leica DM6000 inverted microscope (Leica Microsystems Inc., USA).

### Isolation, Culture, and Characterization of Human Amnion-Derived Mesenchymal Stromal/Stem Cells

Mesenchymal stromal/stem cells were isolated from the amnion of human term placenta of nine healthy donors (aged between 21 and 33 years) within 6 h of birth. Written informed consent and details of the procedure were approved by the Institutional Research Review Board of ISMETT (project identification code: IRRB/18/14). Informed consent was obtained from each donor. Before isolating amnion-derived cells, the amnion was manually separated from the chorion and washed several times with PBS (GIBCO, USA). The membrane was then cut into small pieces and each fragment was decontaminated in: 1. PBS supplemented with 2.5% Esojod (Esoform, Italy); 2. PBS supplemented with 500 U/ml penicillin, 500 mg/ml streptomycin, 12.5 mg/ml amphotericin B, and 1.87 mg/ml cefamezin (Pfizer, Italy); and 3. PBS supplemented with 100 U/ml penicillin and 100 mg/ml streptomycin. Fragmented amnion membrane was incubated for 9 min at 37°C in Hank's balanced salt solution (HBSS) (Lonza, Switzerland) containing 2.5 U/ml dispase (Corning, USA) and then maintained for 5 min at room temperature in RPMI 1640 supplemented with 10% FBS (Thermo Fisher Scientific, USA). Thus, the amniotic fragments were digested with 0.94 mg/ml collagenase A (Roche, Germany) and 20 mg/ml DNase I (Roche, Germany) for 2.5 h at 37°C. Afterward, the digest was filtered using both 100 and 70 μm cell strainers (BD Falcon, USA), pelleted by centrifugation at 300 g for 10 min, and resuspended in RPMI 1640 medium supplemented with 10% FBS for cell counting. Harvested cells were cultured in monolayer in polystyrene culture dishes (Corning, USA) at 37°C and 5% CO_2_ in Chang Medium (Irvine, USA), whereas cell spheroids were maintained in a suspended state (3D) in 6-well ultralow attachment plate (Corning, NY, USA), which facilitates spheroid formations and their maintenance. hAMSC spheroid cultures were grown in DMEM serum-free medium in 5% CO_2_, at 37°C. To analyze phenotype of hAMSCs, single cell suspensions were washed two times with fluorescence-activated cell sorting (FACS) buffer containing FBS and <0.1% NaN_3_ (BD Biosciences, USA). The cells were then incubated on ice for 30 min with diverse fluorochrome-conjugated antibodies (dilution 1:20) against both positive markers (CD90-PE, CD73-APC, and CD13-APC) and negative markers (CD45-APC and HLA-DR-PE) (BD Biosciences, USA), and then analyzed using FACSAria flow cytometer (BD Biosciences, USA).

### Induction of Osteogenic, Adipogenic, and Chondrogenic Differentiation

The differentiation analysis of hAMSCs was done in both 2D and 3D cultures. Spheroids were dissociated into a single-cell suspension and seeded to the culture plates for cell expansion before differentiation assay. To evaluate osteogenic and adipogenic differentiation, cells were grown for 14 days in α-minimum essential medium (α-MEM) supplemented with both 10% FBS and osteogenic and adipogenic supplements, respectively (R&D Systems, USA). Chondrogenic differentiation was analyzed by growing cells in DMEM/F12 medium containing both insulin-transferrin-selenium (ITS) supplement (R&D Systems, USA) and chondrogenic supplement (R&D Systems, USA). A panel of antibodies consisting of anti-hFABP4, anti-hOC, and anti-hACAN was analyzed by immunofluorescence to define the mature phenotypes of adipocytes, osteocytes, and chondrocytes, respectively (R&D Systems, USA). Fluorescence for each antibody was revealed using EVOS™ FL Digital Inverted Fluorescence Microscope (Fisher Scientific, UK), and signal intensities were calculated with ImageJ software.

### Conditioned Media Preparation

To study the potential paracrine effects of hAMSCs in the differentiation of liver organoids, we hypothesized a co-culture of hAMSCs/liver organoids with a ratio of 1:1 (10^6^ cells for each cell type). Thus, hAMSCs were grown in monolayer (2D hAMSC-CM) or in suspension state as organoids (3D hAMSC-CM). Briefly, for CM collection from 2D culture, the cells at the second passage were plated in a 100 mm × 17 mm dish (Nunc, Germany) at 5 × 10^5^ cells/ml in 10 ml of complete DMEM medium with 10% FBS for 2 days until 90% confluence. The medium was then replaced with serum-free organoid differentiation medium (DM), and the cells were maintained for 3 days, and then the medium was collected and used as it is for liver differentiation experiments. For CM collection from 3D culture, the cells were maintained in suspension state in serum-free DMEM medium. After 1 day of culture, we observed initial spheroid formation. The medium was changed with serum-free DM and after 3 days of conditioning, was collected and used as it is for liver differentiation experiments. The supernatant from both cultures was centrifuged, filtered using a 0.2 μm sterile filter, and frozen at −80°C until use.

### Human Liver Organoid Differentiation

Liver organoids were cultured for 7 days in expansion medium (EM) supplemented with bone morphogenetic protein 7 (BMP7, 25 ng/ml) (MedChemExpress, USA). Medium was then changed into the differentiating medium (DM) which consisted of AdDMEM/F12 supplemented with 1% of both N2 and B27, 50 ng/ml EGF (Peprotech, UK), 10 nM gastrin (Sigma, USA), 25 ng/ml HGF (Peprotech, UK), 100 ng/ml FGF19 (Peprotech, UK), 500 nM A83-01 (MedChemExpress, USA), 10 μM DAPT (MedChemExpress, USA), 25 ng/ml BMP7 (MedChemExpress, USA), and 30 μM dexamethasone (MedChemExpress, USA). Differentiation medium was changed every 2–3 days for 13–15 days of culture. Functional analysis was performed in the collected supernatant (24 h after the last medium change) or in whole organoids.

### Gene Expression Analysis

We performed real-time PCR using cDNA as the template in a 20 μL reaction mixture containing SYBR Select Master Mix (Thermo Fisher Scientific, USA) and a specific primer pair for the following genes: *GAPDH, LGR5, EpCAM, KRT19, HNF1*β*, HNF4A, ALB, NTCP*, and *CYP3A4* (Table 1). Briefly, total RNA was extracted with the miRNeasy Mini Kit and treated with DNAse (QIAGEN, Germany). Subsequently, 100 ng of RNA was transcribed with the high-capacity RNA-to-cDNA kit protocol (Thermo Fisher Scientific, USA) to produce single-stranded cDNA. Expression of mRNA was quantified by PCR using StepOnePlus Real-Time PCR System (Thermo Fisher Scientific, USA). *GAPDH* was used as a reference gene for the relative quantification, assessed by 2^−ΔΔCT^ calculation for each mRNA.

**Table 1:** Primer sequences for real-time PCR analysis.

**Gene**	**Forward (5^**′**^-3^**′**^)**	**Reverse (5^**′**^-3^**′**^)**	**GenBank accession ID**	**Amplicon length (bp)**
ALB	ATGCTGAGGCAAAGGATGTC	AGCAGCAGCACGACAGAGTA	NM_000477.7	84
NTCP	ATCGTCCTCAAATCCAAACG	CCACATTGATGGCAGAGAGA	NM_003049.4	111
CYP3A4	TTCCTCCCTGAAAGATTCAGC	GTTGAAGAAGTCCTCCTAAGCT	NM_017460.6	213
LGR5	GAGGATCTGGTGAGCCTGAGAA	CATAAGTGATGCTGGAGCTGGTAA	NM_003667.4	150
EpCAM	CTGGCCGTAAACTGCTTTGT	AGCCCATCATTGTTCTGGAG	NM_002354.3	181
KRT19	CGACTACAGCCACTACTACAC	GGTGGCACCAAGAATCTTGTC	NM_002276.5	60
HNF1β	ATAGCTCCAACCAGACTCACA	AGGCTGTGGATATTCGTCAA	NM_000458.4	312
HNF4A	ACTACGGTGCCTCGAGCTGT	GGCACTGGTTCCTCTTGTCT	NM_178849.3	125
GAPDH	TCAAGAAGGTGGTGAAGCAGG	ACCAGGAAATGAGCTTGACAAA	NM_002046.6	167

### Viability of hAMSCs and Protein Expression Analysis

In order to analyze cell apoptosis/necrosis in hAMSCs grown in serum-free culture conditions, annexin-V/7-AAD assay kits (BD Biosciences, USA) for cell staining were used following the manufacturer's instructions. Their fluorescences were detected using a BD FACSCanto II instrument, and the data were analyzed with BDFACSDiva version 8.0.1 (BD Biosciences, USA). The levels of different cytokines and growth factors in each conditioned medium were determined using magnetic bead technology from Luminex™ with the ProcartaPlex Human Cytokine Chemokine Growth Factor (Affymetrix, USA) according to the manufacturer's instructions. The concentration of each factor was calculated from standard curves.

### Functional Liver Analysis

We examined the functional properties of liver organoids by analysis of both ALB production and CYP3A4 activity. In particular, liver organoids were grown in EM or DM, and culture supernatant was collected 24 h after the last medium change. The amount of ALB was determined using a human Albumin ELISA kit (R&D Systems, USA). To measure CYP3A4 activity, we used luminescence-based assays. Briefly, cultures were differentiated as described above, and then organoids were removed from the matrigel and incubated with the luminescent substrate (3 μM luciferin-IPA) for 1 h at 37°C. Following incubation, cytochrome activity was measured 8 h later using the P450-Glo Assay Kit (Promega, USA) according to the manufacturer's instructions.

### Statistical Methods

All data were analyzed from at least three independent experiments and expressed as mean ± SD. Data from different groups were compared using computerized statistical software (GraphPad Prism 6.0, USA) with the ANOVA test. When ANOVA revealed a *p* < 0.05, the data were further analyzed with Dunnett's *t*-test. Differences were considered statistically significant at *p* < 0.05.

## Results

### Characterization of Human Liver Organoid Culture

**Figure 1:**
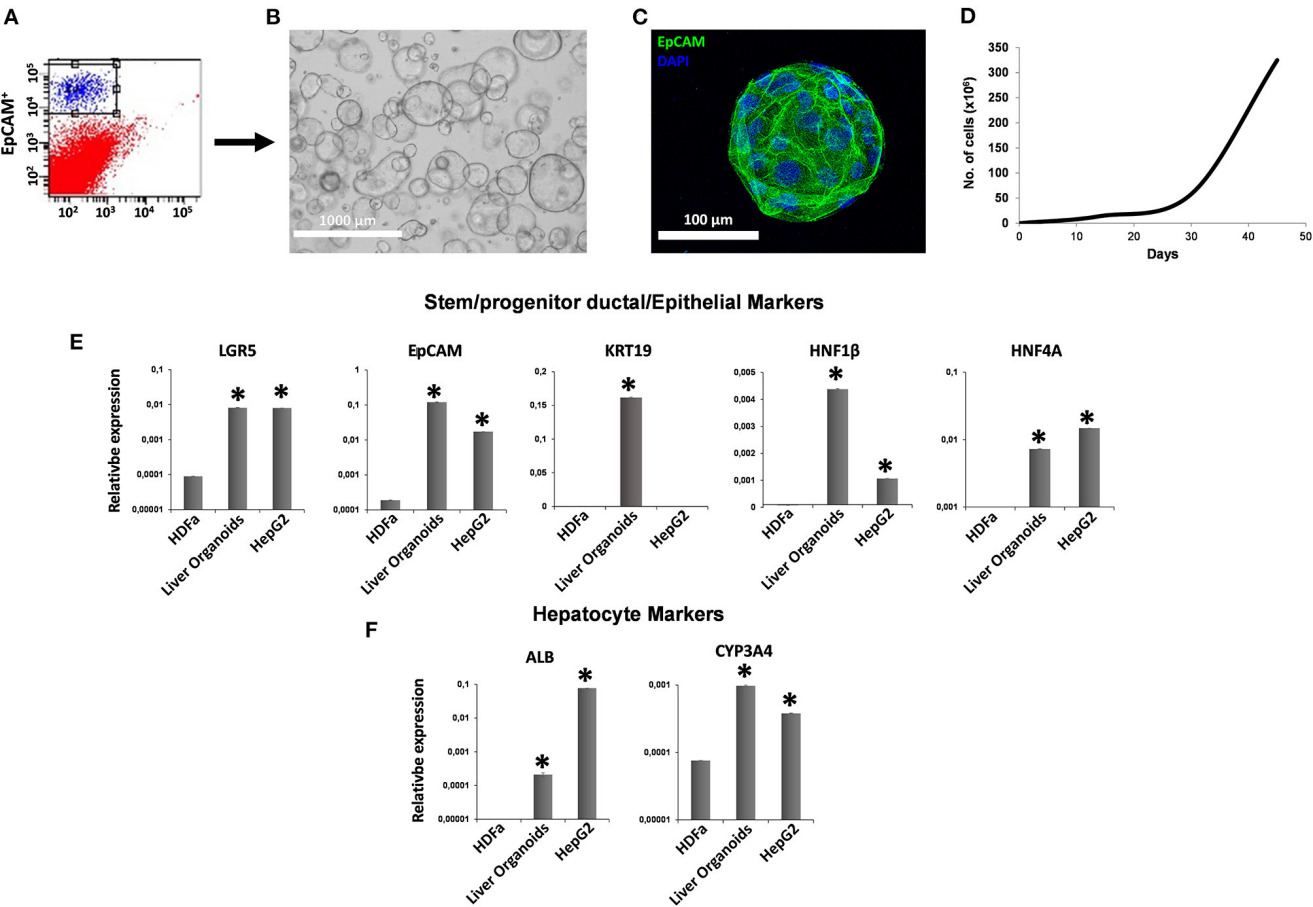
Generation of liver organoids. **(A)** Human liver cell suspensions were analyzed by FACS analysis and separated into EpCAM^+^ and EpCAM^−^ cells. **(B)** Representative DIC image of organoid cultures. **(C)** Immunofluorescent confocal microscopy imaging of liver organoid showing epithelial cell adhesion molecule (EpCAM) (green). **(D)** Number of cells counted per well at each passage from P1 to P5. **(E,F)** Gene expression of stem/progenitor, ductal/epithelial markers, and hepatocyte markers. Transcript levels were normalized to those of GAPDH and expressed as relative expression (2^−ΔCt^). Data are means ± SD. *p < 0.05 vs. HDFa. DIC, differential interference contrast.

After collagenase digestion, we used EpCAM to differentially sort EpCAM^−^ cells from EpCAM^+^ cells (Figure 1A), and we used EpCAM^+^ cells to perform organoid cultures (Figures 1B,C) with a doubling time of ~144.8 h between 30 and 45 days of cultures (Figure 1D). We analyzed the expression of both stem/progenitor and ductal/epithelial markers in organoids. The levels of stem cell marker LGR5 and hepatocyte factor HNF4A were comparable to that of reference cell line HepG2 (21). Meanwhile, epithelial (EpCAM) and ductal (KRT19) markers, as well as hepatocyte factor HNF1β, were more expressed in liver organoids than in HepG2 cells (Figure 1E). Very low expression of functional protein such as ALB and CYP3A4 were observed in liver organoids, with lower ALB expression and higher CYP3A4 expression than in HepG2 cells (Figure 1F).

### Culture, Characterization, and Spheroid Formation of Human Amnion-Derived Mesenchymal Stromal/Stem Cells

**Figure 2:**
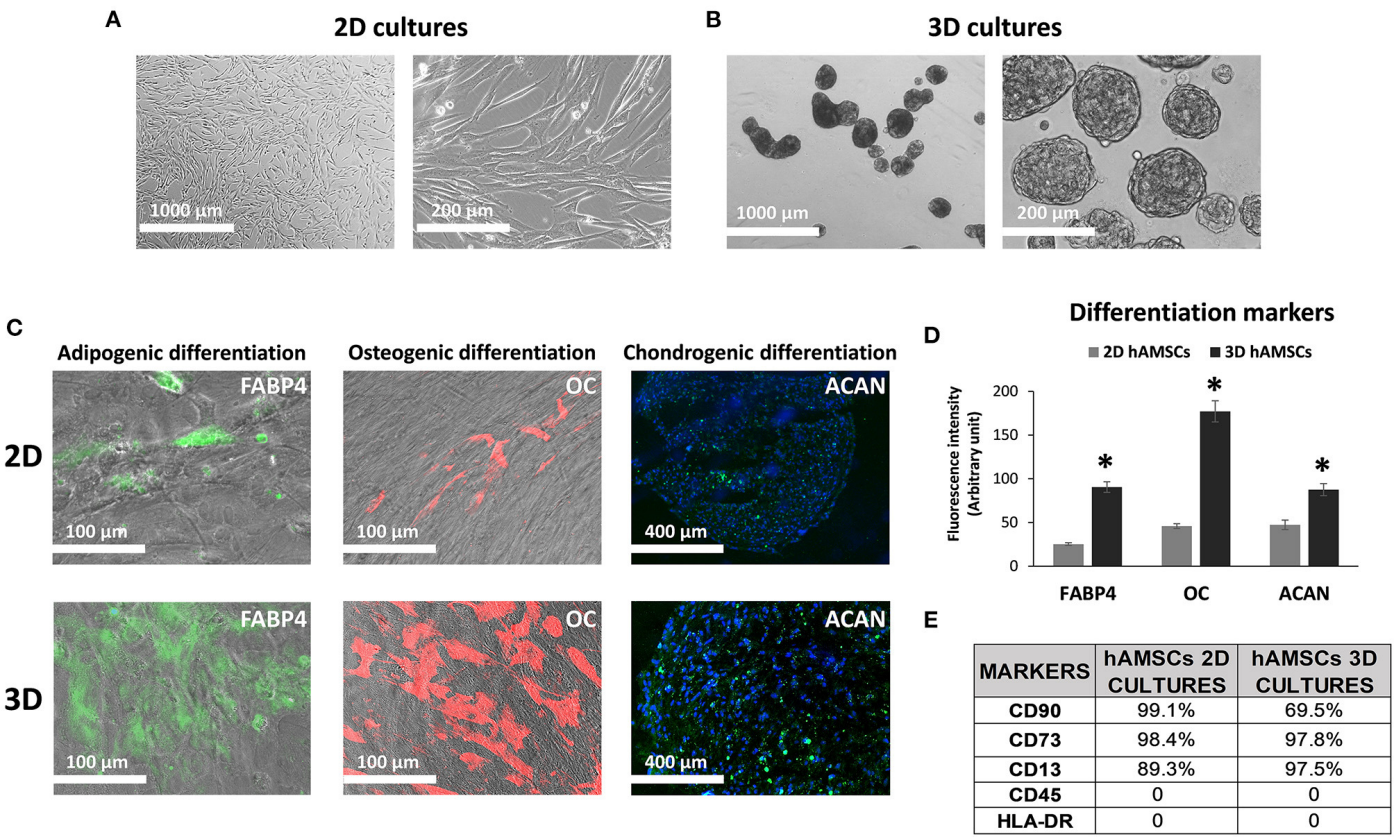
Human amnion-derived mesenchymal stem cells (hAMSCs) cultured as both monolayer and spheroids. **(A)** Representative DIC images of hAMSCs grown in monolayer (2D cultures). **(B)** Representative DIC images of hAMSCs grown as spheroids (3D cultures). **(C)** Immunofluorescence staining localization of fatty acid binding protein 4 (FABP4), Osteocalcin (OC), and Aggrecan (ACAN) in hAMSCs grown as both monolayer and spheroids. **(D)** Graphic depicts FABP4, OC, and ACAN fluorescence intensity in hAMSCs grown as both monolayer and spheroids. **(E)** Cytofluorimetric analysis of the surface marker in hAMSCs grown in 2D cultures and 3D cultures at passage 2. DIC, differential interference contrast. *p < 0.05 vs. 2D hAMSCs.

**Figure 3:**
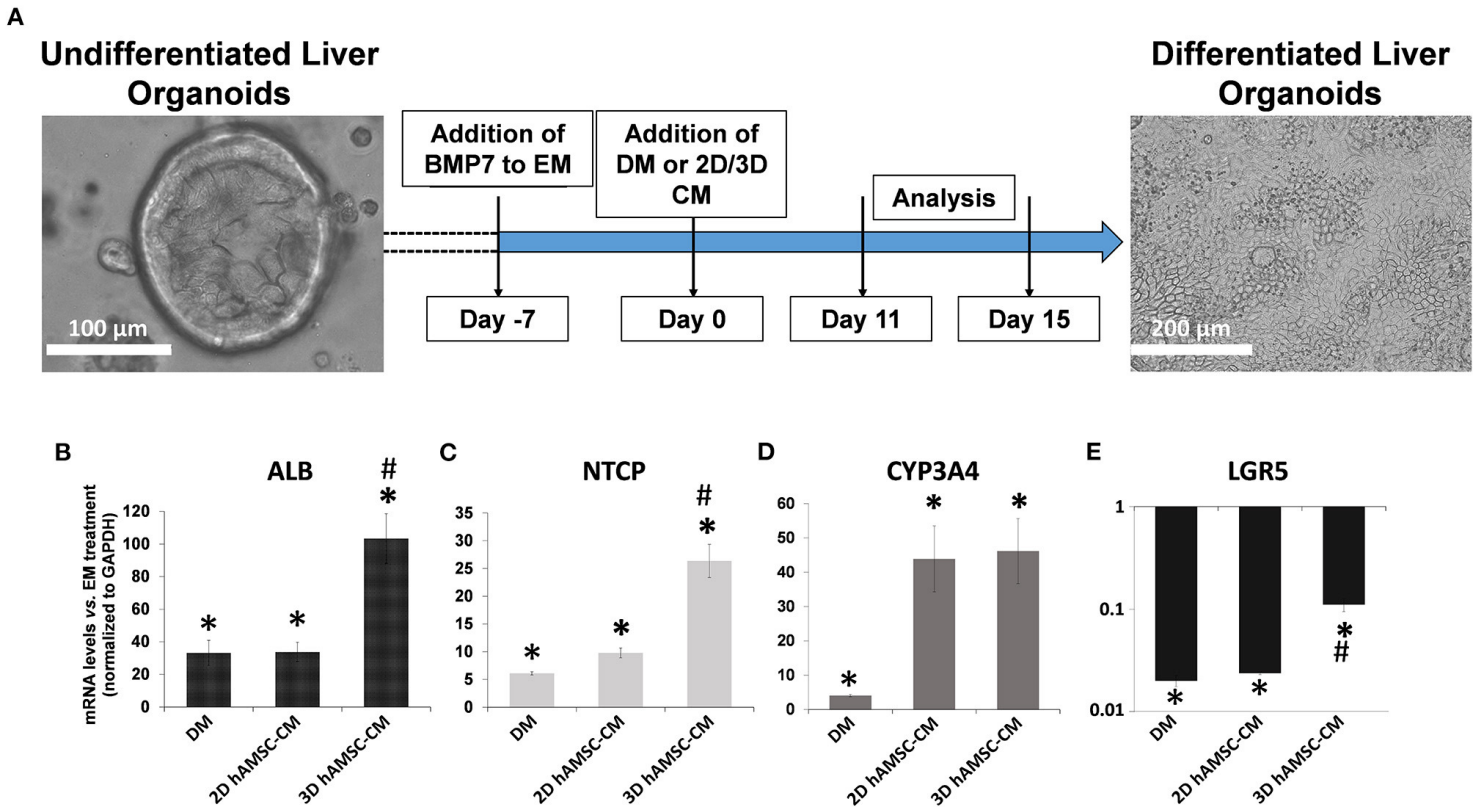
Differentiation of organoids. **(A)** Experimental design (with DIC images of undifferentiated and differentiated liver organoids) to study the potential effects of MSC-derived conditioned medium (CM). **(B–E)** Expression analysis of markers of hepatic differentiation (*ALB, NTCP*, and *CYP3A4*) and of the stem marker (*LGR5*) in liver organoids differentiated for 15 days in each condition. DM, differentiation medium; EM, expansion medium; 2D hAMSC-CM, DM conditioned for 3 days by hAMSCs grown in monolayer; 3D hAMSC-CM, DM conditioned for 3 days by hAMSCs grown as spheroids. Transcript levels were normalized to those of GAPDH and expressed as fold change (2^−ΔΔCt^) vs. EM. Data are means ± SD.; *p < 0.05 vs. EM; ^#^*p* < 0.05 vs. 2D hAMSC-CM. DIC, differential interference contrast.

We maintained hAMSCs in both 2D (Figure 2A) and 3D cultures (Figure 2B). Here, cells spontaneously aggregated and formed compact multicellular spheroids (Figure 2B). To evaluate minimal criteria for defining hAMSCs as mesenchymal stromal/stem cells, cell multipotency was evaluated by the ability of hAMSCs to differentiate into adipocyte-, osteoblast-, and chondrocyte-like cells. Adipogenic, osteogenic, and chondrogenic differentiation processes were detected by immunofluorescence assay of fatty acid binding protein 4 (FABP4), osteocalcin (OC), and aggrecan (ACAN), respectively, in both 2D and 3D hAMSCs cultures (Figure 2C). All three differentiation processes were obtained with hAMSCs and were significantly enhanced when the cells were maintained as spheroids (Figure 2C). In particular, immunofluorescence assay showed an upregulation of FABP4 (3.6-fold), OC (3.8-fold), and ACAN (1.8-fold) proteins in 3D cultures compared to 2D cultures (Figure 2D). Moreover, we also analyzed specific MSCs surface markers in both 2D and 3D cultures. We observed that CD90 (99.1%), CD73 (98.4%), and CD13 (89.3%) were expressed, whereas CD45 and HLA-DR were not expressed in 2D hAMSCs cultures. Moreover, CD90 decreased (from 99.1 to 69.5%) while CD73 increased (from 89.3 to 97.5%) when 2D cultures were compared with 3D cultures (Figure 2E).

### Liver Organoid Differentiation

**Figure 4:**
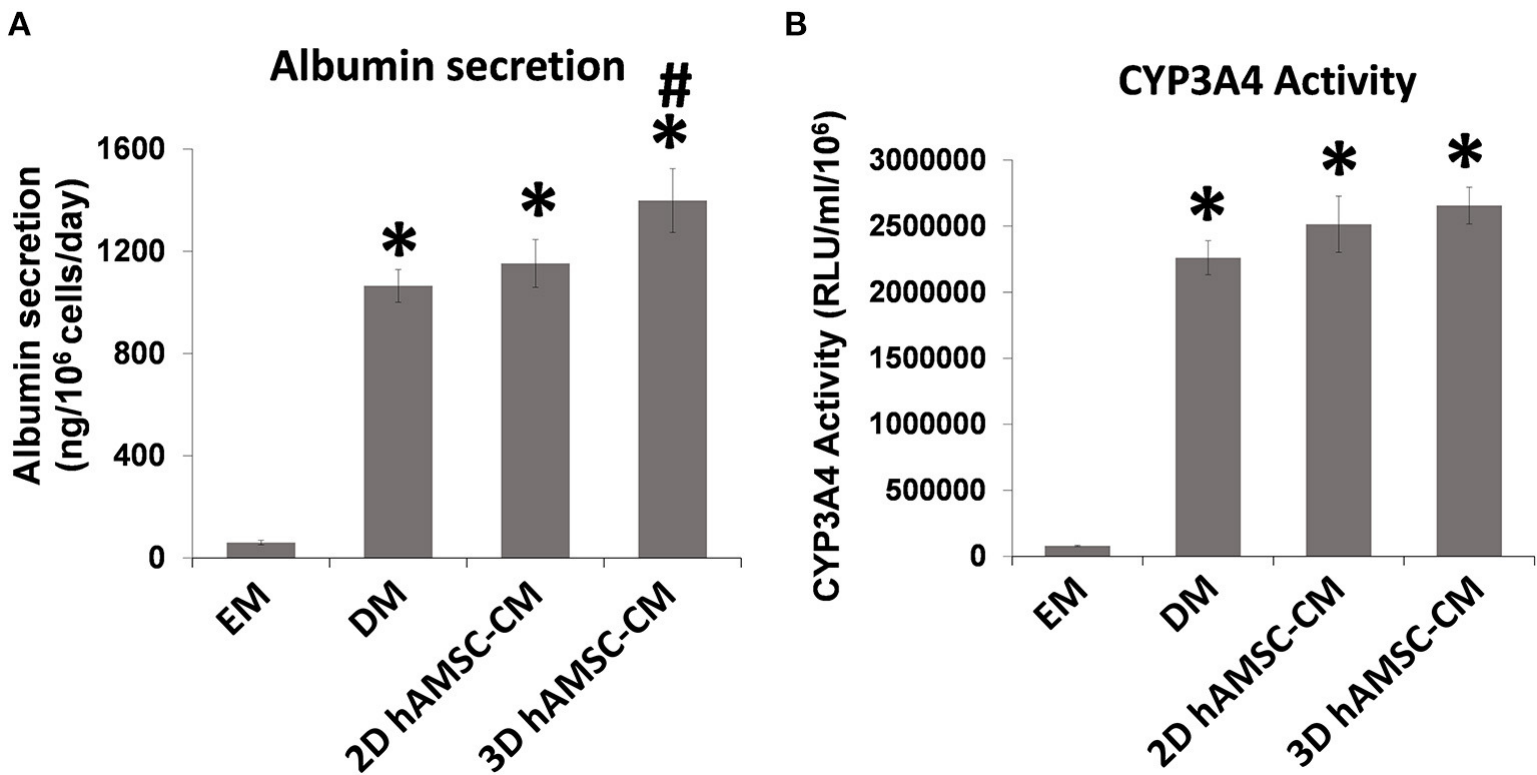
Analysis of **(A)** albumin secretion and **(B)** CYP3A4 activity in human liver organoids differentiated for 15 days in each condition. EM, expansion medium; DM, differentiation medium; 2D hAMSC-CM, DM conditioned for 3 days by hAMSCs grown in monolayer, 3D hAMSC-CM, DM conditioned for 3 days by hAMSCs grown as spheroids. Data are means ± SD. *p < 0.05 vs. EM. ^#^*p* < 0.05 vs. 2D hAMSC-CM.

According to the experimental plan detailed in Figure 3A, we tested the capabilities of undifferentiated liver organoids to produce a functional phenotype. We primed organoids by adding BMP7 to the EM, 7 days before starting differentiation. We then changed EM with differentiation medium (DM, unconditioned or conditioned by both 2D and 3D cultures of hAMSCs), and organoids acquired evident hepatocyte morphologies including polygonal cell shapes (Figure 3A). In all the differentiation conditions, liver organoids showed stronger hepatocyte tracts/functions when compared to the liver organoids grown in EM. Gene expression analysis revealed high expression levels of *ALB, NTCP*, and *CYP3A4* in organoids grown in DM when compared with organoids grown in EM, while *LGR5* significantly decreased in organoids grown in DM (Figures 3B–E). DM conditioned by 2D cultures of hAMSCs (2D hAMSCs-CM) significantly increased the expression of *NTCP* compared to both conventional DM and EM (Figure 3C). DM conditioned by 3D cultures of hAMSCs increased the expression of *ALB, NTCP*, and *CYP3A4* significantly when compared to other treatments (Figures 3B–D). *LGR5* expression was strongly downregulated in both DM and 2D hAMSCs-CM compared to EM while 3D hAMSCs-CM induced a weaker decrease (Figure 3E). Albumin secretion and CYP3A4 activity showed higher levels in DM when compared to EM and this effect was enhanced when DM was conditioned by both 2D and 3D cultures of hAMSCs (Figures 4A,B).

### Spheroid Formation of hAMSCs Increased the Expression of Both Cytokines/Chemokines and Growth Factors

**Figure 5:**
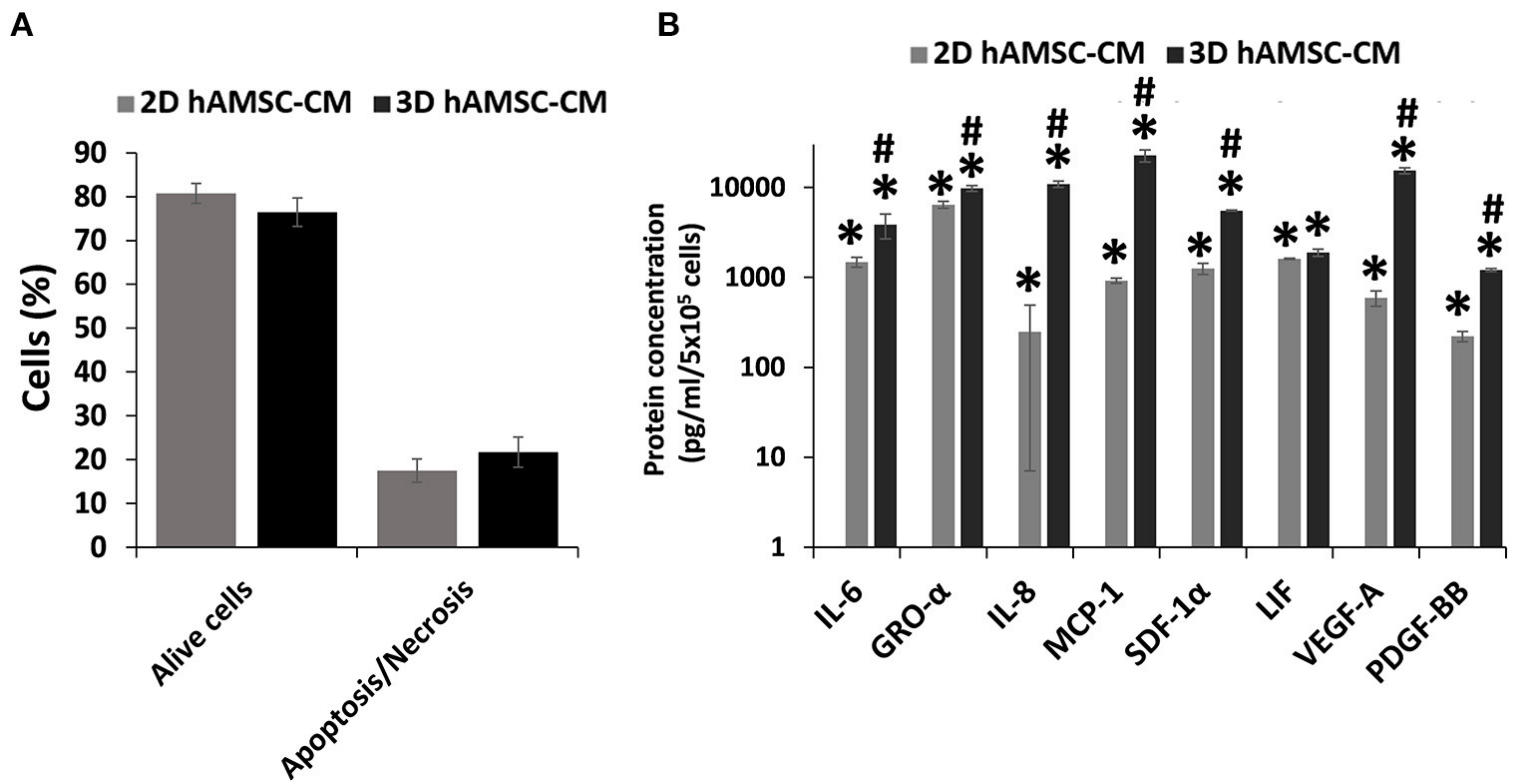
Viability and secretion of cytokines/chemokines and growth factors in monolayer (2D) and spheroids cultures (3D) of human amnion-derived mesenchymal stem cells (hAMSCs). **(A)** Quantification of both alive and necrotic/apoptotic cells in 2D and 3D cultures of hAMSCs. **(B)** The conditioned medium (CM) was collected for both 2D and 3D hAMSCs cultured in differentiation medium (DM) for 72 h and selected cytokines/chemokines and growth factors were analyzed. Data are means ± SD. *p < 0.05 vs. DM. ^#^*p* < 0.05 vs. 2D hAMSC-CM.

Before analyzing the expression of functional factors in the CM, we investigated the viability of hAMSCs when grown under serum-free conditions. Thus, we examined apoptosis/necrosis using double staining with annexin V and 7-ADD. Figure 5A shows that the percentage of viable hAMSCs exposed to serum-free culture was significantly higher in both 2D (80%) and 3D (76%) cultures. We then analyzed the expression of cytokines/chemokines and growth factors, such as IL6, GRO-α, IL8, MCP1, SDF-1α, LIF, VEGF-A, and PDGF-BB, in DM conditioned by both 2D and 3D cultures of hAMSCs. None of these factors was found in the DM, whereas we detected higher levels of those proteins in DM conditioned by both 2D and 3D cultures of hAMSCs, with a greater expression of all factors in CM conditioned by 3D cultures (Figure 5B).

## Discussion

Many studies have reported the therapeutic effects of transplanted MSCs on end-stage liver diseases such as hepatic fibrosis, cirrhosis, and other liver illnesses (22). An improvement of liver function was achieved after injecting autologous MSCs in patients with liver cirrhosis (caused by hepatitis B or C virus, or alcohol) (23). Moreover, some studies showed evidence that the treatment with MSC-derived conditioned medium (MSC-CM) could reduce cell death, upregulate key genes involved in hepatocyte replication, and enhance liver regeneration in a rat model of fulminant hepatic failure (24, 25). Those works demonstrated that MSC-CM has a direct inhibitory effect on death and a stimulatory effect on the proliferation of hepatocytes in *ex vivo*. Another study showed that rats that underwent reduced-size liver transplantation treated with MSC-CM, had significantly lower serum levels of tumor necrosis factor-α (TNF-α) and interleukin-1β (IL-1β) compared with rats that only received the medium treatment (26). Therefore, in recent years the therapeutic efficacy of MSC-derived secretome has been proved (27) and it is believed that MSC-CM contains a number of specific mediators that probably support liver regeneration.

It has been reported that the paracrine therapeutic properties of MSCs can be improved by different preconditioning methods including priming of MSCs with pro-inflammatory cytokine (28), 3D cultures (29, 30), and hypoxia treatment (31). Recently we showed that a 3D culture method is effective for inducing hAMSCs spheroid formation and improving their paracrine activity (32, 33).

Recently, the implementation of the culture method for several organs has been developed, including the intestine, stomach, pancreas, and liver (15, 34, 35). In particular, the long-term maintenance and expansion of liver organoids (derived from EpCAM^+^ primary human liver cells) have been achieved from liver biopsy to form functional hepatic cells both *in vitro* and *in vivo* (15). Epithelial organoids mimic multiple functional aspects of physiological tissues, making them a promising model for testing/studying the right treatment for different human diseases (35).

In our work, we generated undifferentiated liver organoids from EpCAM^+^ liver progenitor cells to study if the paracrine component secreted by human amnion-derived MSCs (hAMSCs) can support liver progenitor cell differentiation. That aspect could consolidate MSCs as a potential regenerative tool that plays a critical role in improving liver repair. We used hAMSCs rather than the widely used bone marrow-derived MSCs (BM-MSCs) because those cells showed similar regenerative effects (33). hAMSCs have stem cell characteristics similar to other adult MSCs, but they also show some embryonic stem cell properties like the expression of pluripotency markers and a higher multilineage differentiation capacity (33, 36, 37). Moreover, the use of neonatal tissues as a source of MSCs has some advantages, such as being easy to obtain a higher quantity without invasiveness and readily cultured to a sufficient number for their use.

Our data showed that undifferentiated liver organoids possess distinct levels of expression of *LGR5, EpCAM, Krt19, HNF1*β*, HNF4A, ALB*, and *CYP3A4* when compared to the reference cell line HepG2 (Figures 1D,E). In order to demonstrate our hypothesis, we performed differentiation of liver organoids and examined it by analysis of expression, production, or activity of markers of mature hepatocytes such as *ALB* and *CYP3A4* (15). We also analyzed the expression of ductal markers, sodium taurocholate cotransporting polypeptide (NTCP), which is expressed in mature hepatocytes and downregulated in proliferating hepatocytes (38). Furthermore, we analyzed *LGR5* expression, a receptor for Wnt agonists (39) that marks adult stem cells in different tissues (40, 41). We tested the effects of hAMSC-CM derived from both 2D and 3D cultures and observed that *ALB* and *NTCP* expressions were significantly upregulated in 3D hAMSC-CM treatment compared with both 2D hAMSC-CM and conventional DM (Figures 3B,C). Moreover, *CYP3A4* expression was upregulated in both 2D and 3D hAMSC-CM compared with DM (Figure 3D). Those data were confirmed by both the over-production of ALB protein and the over-activity of CYP3A4 observed in organoids maintained with 3D hAMSC-CM compared to that in organoids grown with both 2D hAMSC-CM and DM (Figures 4A,B). In order to investigate the molecular mechanisms involved in the above-mentioned effects, we analyzed the secretome produced by both 2D and 3D cultures of hAMSCs.

It has been shown that many cytokines/chemokines play a crucial role in the mechanisms of liver repair/regeneration through their effects on hepatocytes (42). Indeed, it has been demonstrated that growth-related oncogene-α (GRO-α) and MCP1, over-expressed in EPCAM^+^ cells, are potentially involved in the ductular reaction during liver repair after injury (43). Hogaboam et al. revealed that IL-8 agonist was reported to improve liver regeneration and diminish hepatic injury when administered in a mouse model (44). Liepelt and Tacke showed that stromal cell-derived factor-1α (SDF-1α) was constitutively expressed in healthy liver and could contribute to modulate acute liver injury and regeneration (45). In addition, it has been demonstrated that after hepatectomy or liver damage, intense upregulation of IL-6 levels was observed in the liver (46, 47), and IL-6 knockout mice showed impaired liver regeneration (48). Those data highlight the crucial role of IL-6 in liver regeneration. Leukemia inhibitory factor (LIF) is a polyfunctional cytokine that is known to induce acute-phase proteins in the hepatocytes in the liver, and it has been hypothesized that LIF may be involved in the expansion and differentiation of the liver stem cell compartment (49). Franchitto et al. suggested that vascular endothelial growth factor A (VEGF-A) plays an important role in supporting the expansion of hepatic stem/progenitor cell niche by autocrine and paracrine effects on neighboring cells (50). Lou et al. showed that PDGF-BB, belonging to the platelet-derived growth factor (PDGF) family, may play a role in the development and progression of liver fibrosis (51). This PDGF-BB protein was currently indicated as a major inflammatory growth factor playing a central role in the repair process after acute and chronic tissue injuries (52).

In our study, we observed that the above-mentioned cytokines/chemokines and growth factors such as IL6, GRO-α, IL8, MCP1, SDF-1α, LIF, VEGF-A, and PDGF-BB were not contained in conventional DM, while they were significantly over-expressed in hAMSC-CM, with a greater expression of all factors in CM conditioned by 3D cultures. This could explain, at least in part, the greater effect observed with 3D hAMSC-CM on the enhancement of hepatic organoid differentiation. Furthermore, another component of the MSCs secretome, such as MSC-derived exosomes (EXOs), could be involved in mediating the regenerative effects of CM (53). EXOs contain many bioactive molecules that can impact liver regeneration (54, 55) and may also be responsible for the effects of CM observed in our study. Therefore, more research is needed to determine the exact role of EXOs in mediating the effects of hAMSC-CM on liver regeneration.

Liver epithelial cell damage and dysfunctional repair play a role in the development of liver disease, and effective repair likely requires the normal functioning of liver stem/progenitor cells. In this work, we have shown that hAMSCs, principally grown in 3D cultures, are capable of enhancing liver progenitor cell differentiation. In particular, hAMSC-CM was very effective in promoting hepatocyte differentiation, demonstrating that soluble factors secreted by hAMSCs were likely responsible for the result. This work provides evidence of a direct effect of hAMSC-secreted factors on liver progenitor cell differentiation, revealing a potential approach in liver cell transplantation in order to improve this process, with the ultimate goal being efficient organ regeneration.

## Data Availability Statement

The raw data supporting the conclusions of this article will be made available by the authors, without undue reservation, to any qualified researcher.

## Author Contributions

AL and VM conceived and designed experiments, performed cellular experiments, analyzed and interpreted data, and drafted the article. AG and MB analyzed and interpreted data and revised the paper critically. GV, DP, and DG collected and processed liver organoids and performed cellular and molecular experiments. MP collected and processed placenta samples and performed cellular experiments. PC revised the paper critically for important intellectual content. All authors have seen and approved the final draft of the manuscript.

## Funding

This research was funded by UPMC International, Pittsburgh, USA (Project number: I00000216).

## Conflict of Interest

The authors declare that the research was conducted in the absence of any commercial or financial relationships that could be construed as a potential conflict of interest.

## Publisher's Note

All claims expressed in this article are solely those of the authors and do not necessarily represent those of their affiliated organizations, or those of the publisher, the editors and the reviewers. Any product that may be evaluated in this article, or claim that may be made by its manufacturer, is not guaranteed or endorsed by the publisher.
